# Selecting External Controls for Internal Cases Using Stratification Score Matching Methods

**DOI:** 10.3390/ijerph19052549

**Published:** 2022-02-23

**Authors:** Stefanie A. Busgang, Lance A. Waller, Elena Colicino, Ralph D’Agostino, Irva Hertz-Picciotto, Chris Gennings

**Affiliations:** 1 Department of Environmental Medicine and Public Health, Icahn School of Medicine at Mount Sinai, New York, NY 10029, USA; elena.colicino@mssm.edu (E.C.); chris.gennings@mssm.edu (C.G.); 2 Department of Biostatistics and Bioinformatics, Rollins School of Public Health, Emory University, Atlanta, GA 30329, USA; lwaller@emory.edu; 3 Department of Biostatistics and Data Science, Wake Forest School of Medicine, Winston-Salem, NC 27101, USA; rdagosti@wakehealth.edu; 4 Department of Public Health Sciences, University of California, Davis, CA 95616, USA; iher@ucdavis.edu

**Keywords:** stratification score matching, external controls, registry-based

## Abstract

Rare-disease registries can be useful for studying the associations between environmental exposures and disease severity, but often require the addition of a healthy comparison control group. Defining a surrogate control group, matched and balanced on potentially confounding variables, would allow for the comparison of exposure distributions with cases from a registry. In the present study, we assess whether controls selected externally, using stratification score (SS) matching, can serve as effective proxies for internal controls. In addition, we use methyl paraben (MEPB) to compare the estimated associations between an externally matched sample and case–control frequencies in a cohort with internally matched controls. We started with 225 eligible cases of autism spectrum disorder (ASD) from Childhood Autism Risks from Genetics and the Environment (CHARGE), 241 internal controls from CHARGE, and 265 external controls from the National Health and Nutrition Examination Survey (NHANES) cycles 2005–2016. We calculated the SSs using demographic covariates and matched 1:1 using a caliper method without a replacement. The distribution of the covariates and the mean squared error of the paired differences (MSE_paired_) in the SSs between the internal and external group were similar (MSE_paired_ = 0.007 and 0.011, respectively). The association between MEPB and ASD compared to the controls was similar between the externally matched SS pairs and the original frequency matched cohort. Controls selected externally, via SS matching, can provide a comparable bias reduction to that provided by the internal controls, and therefore may be a useful strategy in situations when the internal controls are not available.

## 1. Introduction

National registries of health outcomes, such as cancer, birth outcomes, and HIV patient registries, and the Nonalcoholic Steatohepatitis Clinical Research Network (NASH CRN), provide valuable data resources for studying chronic diseases where the disease of interest is rare and patients may generate clinical data across a long period of time. In the context of environmental research, a national registry additionally provides an opportunity to study the exposure levels in relation to disease severity. However, the precise role of certain environmental exposures may be challenging to determine in this context, because the exposure of interest may be related to the onset of disease itself, the severity, the progression of disease, or any combination of these. Thus, the exposures of interest may be over-represented in such registry databases, relative to the exposure distribution in the broader population, thereby reducing the generalizability of results.

Since registries focus on data related to individuals with the disease of interest, measuring severity of the disease rather than presence alone, one specific challenge in assessing the potential health impacts of environmental exposures in a registry-based analysis is the lack of an obvious parallel control group without the disease. Associations may be missed if chemical exposures are being studied in relation to severity only. There is a need for a comparison group, without the disease of interest, but otherwise comparable to registry cases with regard to variables that are identified as potential confounders, when studying environmental exposures in registry-based studies. The recent increase in consortia of exposure assessment laboratories (and associated public data repositories), such as the Human Health Exposure Analysis Resource (HHEAR) [1], (which is a continuation of the previously funded Children’s Health Exposure Analysis Resource (CHEAR) [2]), provides an opportunity to create a surrogate control group for comparison with a national registry.

Case–control study designs are commonly used when studying rare disease outcomes, because they are better equipped than cohort studies to recruit a sufficient number of patients with the rare disease of interest, often in a much shorter time period [3]. There are four subtypes of case–control studies, each having their own set of benefits and challenges: nested case–control study, case–cohort study, case crossover study, and matching [4]. For the nested case–control and case–cohort studies, the selection of controls is from a random sample that gave rise to the cases, thus reducing the selection bias. When utilizing a consortium as a source of controls, studies are disparate and the controls do not arise under the same conditions, making such designs not logistically feasible. Similarly, the case crossover design is not appropriate for our goal since this type of study design is used for acute events and, in our motivating examples, the disease outcome of interest is often long term. Thus, we focus on matching methods to select cases from a consortium, using overlapping confounders of interest.

In epidemiologic analyses, matching on important covariates can increase efficiency for the confounder control and improve statistical precision [5]. Within case–control studies, there are several methods for matching, including frequency matching, partial matching, full matching and marginal matching [6]. Frequency matching often refers to a case selection protocol based on basic demographic frequency distributions among cases and is easy to implement when few matching factors are identified (i.e., age and sex categories). Since the pool of external controls is not from the same study population that gave rise to cases, additional matching factors beyond just age and sex should be used. Partial and full matching require that a selected control have the same characteristics as the case on certain confounders. For full matching, this can result in a smaller sample size, thus less power, if a case cannot be fully matched to a control. Partial matching has been shown to be similarly efficient to full matching, but less stringent, matching on fewer confounders and allowing a certain distance in differences on continuous confounders between cases and controls [7]. When there are many potential confounders, the use of a scalar variable (i.e., a score summarizing across multiple variables) can allow for better confounder adjustment and marginal matching [8].

More specifically, a stratification score (SS) is defined as the estimated probability of disease, conditional on a set of potential cofounding variables [9]. The SS has some of the same properties as a propensity score (PS) [10], but allows for matching between cases and controls rather than between those who are exposed and those who are not. The goal of matching on SS is to balance the distributions of important covariates between cases and controls, resulting in a more appropriate study base population of controls to select. Some previous studies have used this method for selecting controls from a nested case–control study [11,12]. Though they refer to PS in their methods, we choose to distinguish the terminology and instead use the term SS since some key assumptions of PS cannot be met under our experimental conditions. Specifically, unlike PS, the assumption that the exposure assignment of interest and the case/control outcome are conditionally independent, given the SS (referred to as strongly ignorable treatment/exposure assignment) is not appropriate in our case–control setting since exposures are continuous rather than binary and temporality issues may arise if exposures are measured at the time of outcome assignment. As detailed in the previous literature, a SS is not a panacea for all sources of bias, but can be used as a retrospective balancing score so that the exposure distributions among case–control pairs or groups with similar SS can be directly compared [9].

In the sections below, we build upon the concept of SSs and propose an approach allowing us to add environmental analyses to ongoing registry studies thereby expanding the set of potential research topics. We use the Childhood Autism Risks from Genetics and the Environment (CHARGE) cohort (a frequency matched cohort) to compare a sample of SS matched Autism Spectrum Disorder (ASD) cases, corresponding the frequency matched controls with a sample of the SS matched ASD cases and externally sourced controls from the National Health and Nutrition Examination Survey (NHANES). We demonstrate diagnostics to check for balance of covariates among case and control groups after matches have been selected. To evaluate our approach, we apply the methods to a cohort with existing internal controls to provide evidence that our method can select external controls, balanced on confounder distributions, similar to frequency matching from the beginning of the data collection. We then evaluate the associations between methyl paraben (MEPB) and case–control status comparing the results with frequency matched versus internally matched versus externally matched controls. While the internal controls are helpful in assessing performance of the proposed approach in our example, in practice, we can use the proposed method even in the absence of internal controls.

## 2. Materials and Methods

### 2.1. Demonstration 1

The CHARGE cohort is a large epidemiological case–control study, initiated in 2002. The eligibility criteria required recruited children to be between the ages of 24 and 60 months, born in California, residing in the study catchment areas, living with at least one biologic parent, and having a parent that speaks English or Spanish. Cases of autism were identified through regional centers that contract with the California Department of Developmental Services. Controls within the CHARGE study were identified through state birth files and are frequency matched to the age, sex, and broad residential catchment area distribution of the autism cases. For those children who enrolled in the study, the diagnostic group was confirmed through standardized clinical assessments at the UC Davis Medical Investigation of Neurodevelopmental Disorders Institute. At this clinical visit, urine samples were collected from both cases and controls for environmental exposure analysis. Additional details of the study are presented elsewhere [13].

NHANES is a nationally representative cross-sectional dataset, collected by the National Center for Health Statistics. Using a complex multistage probability sampling design, NHANES recruits over 5000 Americans of all ages each year. Surveys are provided in English or Spanish [14]. We include the 2005–2016 NHANES cycles in the current analysis to overlap with the recruitment years for CHARGE. Due to the lack of measures regarding neurodevelopmental status and the low prevalence of ASD, we assume that all children in this cohort are not diagnosed with autism; thus, they are characterized as controls. Urine specimens are collected on the same day as the collection of demographic self-reported data.

In our comparisons below, the internal sample refers to cases from CHARGE and controls from CHARGE. There were 231 diagnoses of ASD and 248 controls evaluated as a subset that was selected for a CHEAR project, based on the availability of biologic specimens for analysis of specific environmental exposure-related chemicals (to be reported elsewhere). The external sample refers to the same 231 cases of ASD from CHARGE and a subset of controls sampled from NHANES. Participants between the ages of 2 and 6 years from the 2005–2016 NHANES cohorts were combined, resulting in a sample size of 7110 potential controls. Among those, 536 potential external controls were eligible for matching because they were part of the subsample that NHANES randomly selects for biospecimen collection for laboratory evaluation of several chemical classes and specifically had methyl paraben measured. Note, only the 2005–2006 cycle assessed the biological specimens in children under 6 years old; thus, most eligible controls from NHANES are 6 years old. Figure S1 provides a flowchart of participant selection with inclusion and exclusion criteria.

We concatenated the CHARGE and NHANES datasets to estimate the SS for all participants in the external sample with a logistic regression model. For the internal sample, the SSs were estimated from a logistic model, using only the CHARGE cohort. The score represents the conditional probability of being a case, given the following set of covariates—the child’s age in months, year of birth, sex, race, maternal education, birthplace of mother, maternal age, and parental homeowner status. To increase the compatibility between NHANES and CHARGE, we harmonized selected variables to better define overlap, e.g., relating to category definitions (Table S1). For example, the child’s race was categorized as White non-Hispanic, non-White non-Hispanic, and Hispanic in CHARGE to ensure sufficient cell sizes for this analysis. Alternatively, NHANES has 5 categories for race, which required the collapsing and re-defining of the final categorizations. Cases and controls were randomly sorted and then matched to a control using a 1:1 caliper algorithm, without replacement [15]. The recommended caliper width differs in the PS literature [16,17]; here we follow the recommendation of 0.25 of the pooled standard deviation of the logit of the PS [18]. In our case, 0.25 of the pooled standard deviation of the logit of the SS is 0.22. We increased the caliper to 0.30 to retain more cases in the external sample, while still maintaining precision; i.e., a control must have a stratification score no more or no less than 0.30 from the stratification score of its matched case. The publicly available SAS macro %PSMatching was used for our SS matching [19].

After the pairs were matched, unmatched participants were summarized then discarded from further analysis. The closeness of SS within a pair and the distribution of covariates between the cases and controls were assessed for both the internal and external samples. We define the mean squared error of paired differences (MSE_paired_) as:

(1) $${\mathrm{MSE}}_{\mathrm{paired}}=\frac{\sum {\left({\hat{\mathrm{SS}}}_{\mathrm{case}}-{\hat{\mathrm{SS}}}_{\mathrm{control}}\right)}^{2}}{\mathrm{number}\;\mathrm{of}\;\mathrm{pairs}}$$

The covariate balance, before and after matching, was assessed by primarily by standardized differences [20] and visually displayed as a Love plot [21,22]. Additionally, the *p*-values of *t*-tests for the continuous variables and chi-squared tests for dichotomous variables were compared between pre- and post-matched samples; however, since *p*-values are affected by sample size, they are considered a secondary measure to assess that balance is achieved.

All statistical analyses were conducted with SAS statistical analysis software version 9.4 (Cary, NC, USA).

### 2.2. Demonstration 2

We assessed the associations of creatinine-adjusted MEPB on the risk of ASD compared to the controls, in relation to three conditions: the internally matched sample, and the externally matched sample, and the entire CHARGE cohort consisting of frequency matched cases and controls (referred to as the frequency matched sample). MEPB was natural log transformed due to right skewed data and to avoid potentially influential outliers. For the frequency matched sample, we used unconditional logistic regression, controlling for child’s age in months, year of birth, sex, race, maternal education, birthplace of mother, maternal age, and parental homeowner status. For the internally and externally matched samples, we used conditional logistic regression. We selected MEPB because increasing the concentrations in urine was associated with higher odds of ASD compared to controls, specifically in this frequency-matched cohort, and thus it was a good candidate for comparison between our variously selected controls [23].

## 3. Results

### 3.1. Demonstration 1

**Figure 1:**
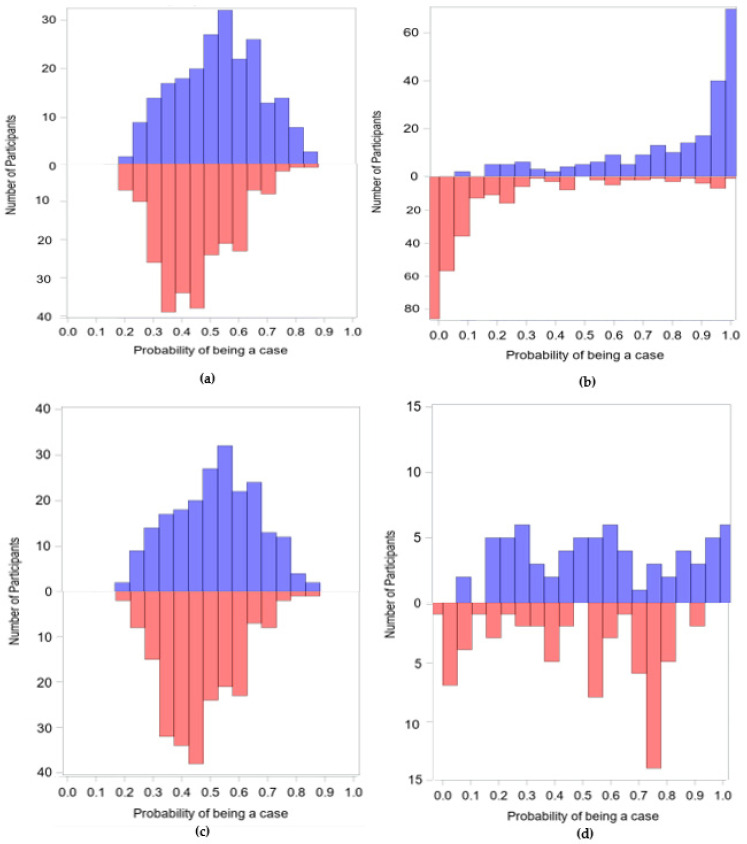
Distributions of SS between cases (purple) and controls (pink) (**a**) before matching using the internal controls, (**b**) before matching using external controls, (**c**) after matching using the internal sample, and (**d**) after matching using the external controls.

Prior to matching, 225 cases and 241 controls remained in the internal sample and 225 cases and 265 controls remained in the external sample after removing participants missing covariates. Pair-wise matching resulted in 216 and 71 pairs for the internal and external samples, respectively. Distributions of the SS, stratified by case status, for each analytic sample before and after matching appear in Figure 1. Prior to matching, controls from NHANES have SSs that are more skewed towards a low probability of being a case, whereas controls in CHARGE had SSs indicating a higher probability of being a case. However, the post-matching distributions of SS are similar for both samples (Figure 1).

**Figure 2:**
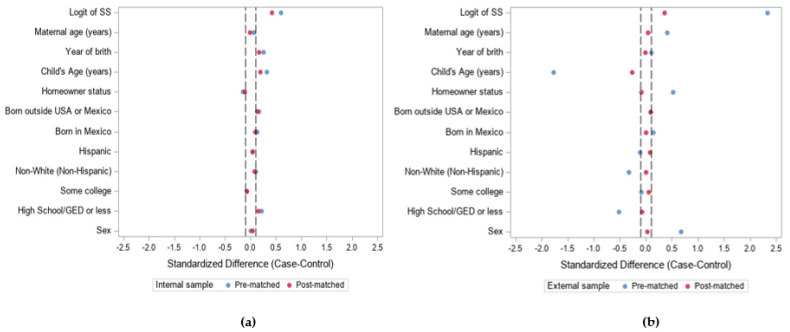
Pre- and post-matching standardized differences of individual covariates between case and control groups for (**a**) the internal sample and (**b**) the external sample. The dashed lines represent the suggested threshold standardized difference proposed by Normand et al. (2001) of less than 0.10 [22,24]. Abbreviations: SS = Stratification score; U.S.A. = United States of America; GED = general education diploma.

Table 1 and Table 2 show the distributions of covariates among cases and controls for the internal and external samples, before and after participants were matched. In comparison to the NHANES controls, CHARGE cases and controls have a higher percentage of boys because ASD affects far more boys than girls and internal controls were frequency-matched on projected sex ratios of cases. In addition, mothers from CHARGE tended to be more highly educated in comparison to NHANES mothers. There are also a higher percentage of children from CHARGE whose parent is a homeowner, used as an indicator of higher socioeconomic status. The Love plot indicates that there is little, if any, benefit from the 1:1 matching for the internal sample (Figure 2). Similarly, post-matched *p*-values indicate that some covariates become more balanced after matching as indicated by an increasing *p*-value, but, overall, there is not an obvious improvement in covariate balance (Table 1). In contrast, the improvement in balance of covariates was enormous for the external sample, e.g., child’s age, sex, ethnicity, education, and home ownership. The Love plot clearly shows an improvement in the distribution of covariates after the participants were matched, with all post-matched covariates closer to a standardized difference of zero (Figure 2) Likewise, all covariates in the external sample appear to not be significantly different between the cases and controls or are at least becoming more balanced (*p*-values increasing; Table 2). Thus, we find the SS approach is successful in increasing the balance for all the covariates in the external sample.

**Figure 3:**
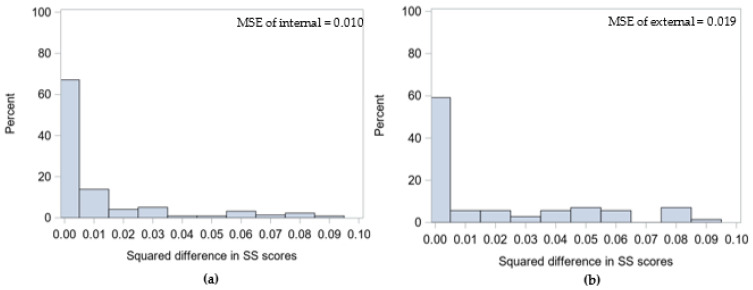
The distribution of error in matched pairs for (**a**) the sample using internal controls (N = 216 pairs) and (**b**) the sample using external controls (N = 71 pairs) using the caliper method with a 0.30 caliper. Abbreviations: SS = stratification score; MSE = mean squared error.

The MSE is small for both the internal and external samples (0.007 and 0.011, respectively). The squared difference in the SS between cases and external controls is similar in magnitude to the squared difference of internally matched pairs (Figure 3). However, information is lost in both samples due to the cases being dropped, either because there are too few controls or there were no controls to be matched to cases with the highest SS.

### 3.2. Demonstration 2

The associations between the log-transformed MEPB and risk of ASD compared to controls were assessed in the frequency, internally, and externally matched samples. The frequency matched sample serves as the “truth” or gold standard since distributions of important covariates were close to the balance by design. We found a significant association between MEPB and ASD, so that for every 1 unit increase in the log-transformed MEPB concentrations, there was 1.20 times higher odds of developing ASD (*p* < 0.001; Table 3). The association between MEPB and ASD remained significant in the internal sample, with a slight reduction in magnitude (OR = 1.15, *p* = 0.005). Lastly, although the external matched sample did not result in a significant association, the magnitude of the OR (OR = 1.16, *p* = 0.08) was similar to those of the frequency and internal samples.

## 4. Discussion

Matching on estimated SSs allows for the comparison of exposure distributions from case–control pairs, where each member has a similar probability of being a case. Here, we determined that externally matched controls can be as similar to cases as internal controls, based on a scalar matching variable. The evaluation of the pre- and post-matched samples shows the post-matched controls appear to be more similar, on average, to the cases, across covariates compared to pre-matched. For example, there are 19% girls and 81% boys in the case group in our example. As one might expect from a more general sample, prior to matching, our external control group was 55% girls and 45% boys. After matching on SS, post-matched controls contained 30% girls and 70% boys. We found the MSE for the externally controlled matched sample to be similar to the MSE for the internally controlled sample. Almost 80% of case–control pairs in the internal sample and almost 70% of pairs in the external sample had less than a 0.1 difference in SS; this demonstrates the effectiveness of our matching algorithm. The internal sample did not show a high degree of improvement in balance as shown by small changes in the standardized differences between pre- and post-matched samples (Figure 2a). The minimal improvement in the balance of covariate distributions after post-matching can be attributed to the effectiveness of frequency matching in the original study design.

Almost all cases were maintained using internal matching (n = 9 dropped from post-matched sample), compared to more than half of the cases dropping out post-matching with the external sample. For our example, we used one-to-one matching without replacement; however, our strategy can be expanded to account for more complex sampling schemes, e.g., incorporating clustering effects wherein the SS algorithm first searches for a control match within the same cluster; if a match cannot be found, it moves to a different cluster [25]. In a consortium, this can be applied by looking for a match within the same cohort and using external controls, only when a match cannot be found internally. When there is not a high overlap between SSs, we caution against this method due to a reduction in generalizability.

Previous studies using the CHARGE cohort applied survey weights to account for the differential probabilities of enrollment for case and control groups, such as the differences in maternal education levels, insurance payment type at birth, regional center, parity, and maternal birthplace, comparing participants with those that did not participate in the study [26]. In the current study, we did not apply survey weights and thus may be underrepresenting characteristics of the non-participants. However, compared to the internal sample, there is a slightly higher representation of less educated mothers, which were under represented in those that participated in CHARGE overall, hence likely in the internal sample. Future work will address the scenarios that could benefit from the use of survey weights.

When exposure data are available, we would perform a *t*-test of the exposure means among the final groups of cases and controls or a two-sample Kolmogorov–Smirnov test to measure the difference in distributions between groups. Alternatively, we can perform conditional logistic regression on the matched pairs. Here, we chose conditional logistic regression to assess associations of MEPB with ASD in our SS matched samples and compared the results to those found in an unconditional logistic regression model of the frequency matched sample. Though the effect estimate was only borderline significant in the external sample, we noted the estimated effect was very close in magnitude to that obtained using internal controls. Due to the sample reduction from 447 participants in the frequency matched to 142 participants (71 pairs) using the externally matched controls, we did not have enough power to reach significance for the anticipated effect size (i.e., that obtained using internal controls). This result emphasizes the importance of statistical power and obtaining a sufficient number of matches. Though we started with a large pool of external controls from the NAHNES cycles 2006–2015, the necessary variables for our example included a biologic specimen with a result for a specific analyte, which led to a major drop in sample size (Figure S1).

The pool of potential controls may be limited by the overlap in important variables (e.g., maternal education); however, there is often a standard set of covariates collected across (almost) all epidemiologic studies, including basic demographics, such as age, race, and sex. Often, there are additional common variables between studies, such as body mass index (BMI) and parental education. One advantage of the studies included in a consortium is the larger pool of potential controls relative to cases, allowing for analysts to specify required criteria that are dependent on the analysis of interest. Additionally, a consortium, such as HHEAR, which measures environmental exposures typically also has quality assurance programs whereby the same quality control samples are run across all studies and labs, increasing the ability to harmonize laboratory measures across disparate studies [27].

Model-based matches are only as good as our SS and such score-based adjustments are not guaranteed to address all possible sources of bias. For example, the location of the participant is often important in environmental research with local concentrations of confounder values driving the local bias. Since exposure distributions and disease rates can vary widely by location and this information may not be provided in a publicly available consortium, we propose repeating our matching algorithm many times by randomly re-ordering the cases, resulting in different sets of controls being selected. This pseudo-randomization increases the variability in exposures that are implicit within a pair, even if the SSs are the same. A similar methodology was used in PS matching and is suggested only when matching is performed without replacement, which is likely the same for SS matching [28].

Here, we apply the PS framework to a scenario that is different than the traditional binary exposure assignment or randomized (or non-randomized) control setting, and thus assumptions from the PS may not apply to SS. The goals of PS are primarily to establish causal inference by controlling for factors that temporally occurred prior to the exposure assignment [10]. SS matching does not intend to establish the same causality as PS because the study designs to which one may apply SS can vary and may not be able to determine such temporality. With a high level of confidence, it is typically agreed that sex of the child satisfies temporality, and usually parental educational attainment would precede most exposures of interest. However, a variable, such as BMI, may both influence and be influenced by cumulative environmental exposures. Similarly, environmental exposures may have a critical window of effect [29]. For example, in utero exposures may affect factors that precede the current assessment of exposure by a long period, which again makes it challenging to select covariates based on the same temporality principles as PS. In addition, exposures may not occur at one time point; instead, exposures may occur over long periods, resulting in cumulative effects, or may be persistent (e.g., polyfluoroalkyl substances), indicating an exposure that may or may not be recent. These features of environmental exposure measurements provide an important context for interpreting our results. Biological samples were collected at the same time as the clinical assessment and diagnosis in this example assessing MEPB on ASD. For this outcome, therefore, traditional goals for PS methods would not be appropriate since balancing basic demographics would not establish temporality of exposure and disease, making causal inference indeterminate. However, here, our goal is to use SS matching to select comparable populations. Using CHARGE, we are able to select a group of controls that are comparable on characteristics and then we can evaluate whether exposure levels vary between these groups. Essentially, SS serves as a more flexible frequency matching tool that can account for more complex relationships between the baseline characteristics. Although we did not study these types of relationships here, we could extend the approach to include interaction terms and higher order terms when building our SS models to attain comparability at a level that frequency matching itself may be unable to do. Furthermore, PSs estimate the probability of exposure and observe how exposure predicts disease, whereas SSs estimate the probability of disease-given covariates to observe if people with similar backgrounds, only differing on having disease or not, have the same exposure.

While promising, our proposed SS approach merits further development and assessment. A fundamental assumption of using a surrogate control group is that the source of controls is disease-free. In our example, NHANES does not assess ASD, so we had to assume that all NHANES participants are disease-free. There is likely some non-differential misclassification of the outcome in Demonstration 1, so that cases are misclassified as controls, which will tend to bias the results toward the null. When possible, potential cases should be removed. For example, NHANES asks about cognitive status among older adults. If there were questions about neuro-cognition among children, we could remove the participants who are suspected of having ASD. In scenarios when the disease of interest is very rare, as is the case with ASD, the assumption of disease-free external controls is reasonable based on low probability of having ASD.

In addition, it is important to ensure that all covariates either remain balanced or become more balanced after matching. If some covariates become more imbalanced after matching, the analyst may need to re-think the variables included in the SS model. In a randomized trial, the assumption is that all baseline covariates should be balanced between groups after randomization, including the measured and unmeasured covariates as well as the covariates associated with the outcome or not, although small trials can easily go awry of this balance. Therefore, when identifying matches using the SS model, all the measured covariates should be balanced after matching, regardless of whether they were balanced prior to matching. Matched data, created so that all the covariates are balanced after matching, can be applied to multiple exposures if the covariates selected are not specific only to the original exposure of interest.

## 5. Conclusions

SS methods provide a novel tool for selecting a surrogate external comparison group that is similarly balanced on covariates as an internal control group. The approach utilizes the increased accessibility of both registry and large-scale public datasets with relevant exposure measures and matching variables. Typical rare disease registries may not be designed to assess the effects of environmental exposures and hence may not recruit an internal control group, providing a challenge that can be addressed with the proposed approach. The CHARGE cohort provided a unique opportunity to determine the baseline similarity between internal cases and our proposed external controls for comparison. The examination of the post-matched groups for both samples indicates balance on almost all covariates. Balance provides the support that the observed differences in exposure distributions are related to the disease rather than confounding.

The second demonstration showed that our matching method, using external controls, resulted in an effect estimate of a remarkably similar magnitude to that of the frequency-matched and internally matched samples, despite the sample size reduction, which widened the confidence intervals. Though the estimate was not significant at the 5% significance level, due to the reduction in power, the close estimated effect sizes provide evidence that our strategy works. The challenge, then, of this method is to identify a large enough pool of eligible external controls to achieve the desired statistical power.

Together, these demonstrations provide support for the use of SSs in identifying controls (e.g., participants pooled in a consortium) to match cases (e.g., participants identified in a disease registry) to answer research questions that could not be answered within a cohort of cases alone.

## Figures and Tables

**Table 1:** Demographic characteristics (mean (SD) or frequency (%)) of the cases and controls in the internal sample, before and after matching with a 0.30 caliper using internal controls.

	Before Matching			After Matching		
	Cases (N = 225)	Controls (N = 241)	*p*-Value ^a^	Cases (N = 216)	Controls (N = 216)	*p*-Value ^a^
Age of child (years)	4.07 (0.72)	3.83 (0.75)	<0.001	4.05 (0.73)	3.91 (0.71)	0.043
Year of birth	2007.26 (3.22)	2006.47 (2.91)	0.006	2007.19 (3.24)	2006.67 (2.91)	0.081
Maternal age (years)	30.74 (5.76)	30.37 (5.39)	0.476	30.60 (5.69)	30.63 (5.40)	0.952
Sex						
Female (ref)	43 (19%)	46 (19%)	0.995	39 (18%)	43 (20%)	0.624
Male	182 (81%)	195 (81%)		177 (82%)	173 (81%)	
Maternal education						
Bachelor/graduate/professional (ref)	109 (48%)	129 (53%)	0.310 0.003	108 (50%)	113 (52%)	0.312 0.038
Some college	73 (32%)	89 (37%)		70 (32%)	80 (37%)	
High school/GED or less	43 (19%)	23 (10%)		38 (18%)	23 (11%)	
Child’s race						
White (non-Hispanic) (ref)	107 (48%)	132 (55%)	0.161 0.652	105 (49%)	118 (55%)	0.355 0.584
Non-White (non-Hispanic)	55 (24%)	46 (19%)		52 (24%)	44 (20%)	
Hispanic	63 (28%)	63 (26%)		59 (27%)	54 (25%)	
Birth place of mom						
U.S.A. (ref)	166 (74%)	205 (85%)	0.081 0.022	163 (75%)	181 (84%)	0.208 0.104
Mexico	18 (8%)	10 (4%)		15 (7%)	9 (4%)	
Outside the U.S.A. or Mexico	41 (18%)	26 (11%)		38 (18%)	26 (12%)	
Homeowner status						
No (ref)	73 (32%)	58 (24%)	0.044	68 (31%)	54 (25%)	0.135
Yes	152 (68%)	183 (76%)		148 (69%)	162 (75%)	
Stratification score	0.53 (0.15)	0.54 (0.09)	<0.001	0.52 (0.14)	0.46 (0.12)	<0.001

^a^*p*-values provided for either a two-sample *t*-test for continuous variables or a chi-square for categorical variables when comparing the demographics between cases and controls. Abbreviations: U.S.A. = United States of America; GED = general education diploma; ref = reference.

**Table 2:** Demographic characteristics (mean (SD) or frequency (%)) of the cases and controls in the external sample, before and after matching with a 0.30 caliper using external controls.

	Before Matching			After Matching		
	Cases (N = 225)	Controls (N = 265)	*p*-Value ^a^	Cases (N = 71)	Controls (N = 71)	*p*-Value ^a^
Age of child (years)	4.07 (0.72)	5.52 (0.90)	<0.001	4.44 (0.52)	4.69 (1.21)	0.112
Year of birth	2007.26 (3.22)	2006.87 (4.12)	<0.001	2008.15 (3.14)	2008.21 (5.09)	0.937
Maternal age (years)	30.74 (5.76)	28.04 (7.56)	<0.001	29.92 (6.37)	29.69 (6.73)	0.838
Sex						
Female (ref)	43 (19%)	145 (55%)	<0.001	21 (30%)	22 (31%)	0.855
Male	182 (81%)	120 (45%)		50 (70%)	49 (69%)	
Maternal education						
Bachelor/graduate/professional (ref)	109 (48%)	43 (16%)	0.256 <0.001	25 (35%)	24 (34%)	0.730 0.573
Some college	73 (32%)	99 (37%)		28 (39%)	26 (37%)	
High school/GED or less	43 (19%)	123 (47%)		18 (25%)	21 (29%)	
Child’s race/ethnicity						
White (non-Hispanic) (ref)	107 (48%)	63 (24%)	<0.001 0.025	23 (32%)	26 (37%)	1.000 0.593
Non-White (non-Hispanic)	55 (24%)	112 (42%)		23 (32%)	23 (32%)	
Hispanic	63 (28%)	90 (34%)		25 (25%)	22 (31%)	
Birth place of mom						
U.S.A. (ref)	166 (74%)	217 (82%)	<0.001 <0.001	53 (75%)	56 (79%)	1.000 0.494
Mexico	18 (8%)	10 (4%)		5 (7%)	5 (7%)	
Outside the U.S.A. or Mexico	41 (18%)	38 (14%)		13 (18%)	10 (14%)	
Homeowner status						
No (ref)	73 (32%)	167 (63%)		35 (49%)	31 (44%)	
Yes	152 (68%)	98 (37%)	<0.001	36 (51%)	40 (56%)	0.501
Stratification score	0.81 (0.24)	0.16 (0.24)	<0.001	0.57 (0.27)	0.49 (0.27)	0.060

^a^*p*-values provided for either a two-sample *t*-test for continuous variables or a chi-square for categorical variables when comparing demographics between cases and controls. Abbreviations: U.S.A. = United States of America; GED = general education diploma; ref = reference.

**Table 3:** Association of MEPB and ASD compared to the controls among three conditions.

	OR (95% CI)	*p*-Value
Frequency matched (N = 466 total participants)	1.20 (1.09, 1.33)	<0.001
Internally matched (N = 216 pairs)	1.15 (1.04, 1.27)	0.005
Externally matched (N = 71 pairs)	1.16 (0.98, 1.37)	0.080

## Data Availability

The data from the CHARGE cohort presented in this study are openly available in the HHEAR Data Repository at 10.36043/1461_219 and 10.36043/1461_222. Datasets relating to NHANES participants are also publicly available and the data can be found here: https://wwwn.cdc.gov/nchs/nhanes/Default.aspx (accessed on 13 September 2021).

## Supplementary Materials

The following supporting information can be downloaded at: https://www.mdpi.com/article/10.3390/ijerph19052549/s1, Figure S1. Flowchart of participant inclusion and exclusion criteria for CHARGE and NHANES samples; Table S1. Table demonstrating the covariates used in the SS model and how the variables were measured in individual studies and re-categorized for the current analysis.
