# Supplementary material for: Selecting External Controls for Internal Cases Using Stratification Score Matching Methods
Source: Int J Environ Res Public Health. 2022 Feb 23;19(5):2549. doi: 10.3390/ijerph19052549 (PMC8909853; doi:10.3390/ijerph19052549)

**Table S1.** Table demonstrating covariates used in SS model and how variables were measured in individual studies and re-categorized for the current analysis.

| Variable | CHARGE | NHANES | Re-categorization |
|---|---|---|---|
| Maternal Education | Mom's education<br>1. High school diploma/GED or less<br>2. Some college (incl. vocational. 2-yr degree)<br>3. Bachelor's degree<br>3. Graduate or professional degree | Female household ref's education<br>1. Less than 9th grade<br>1. 9-11th Grade (no diploma)<br>1. High school Grad/GED equivalent<br>2. Some college or AA degree<br>3. College graduate or above | 1. High school diploma/ GED or less<br>2. Some college<br>3. College graduate or above |
| Child's Race | Child's Race<br>1. White (non-Hispanic)<br>2. Non-White (non-Hispanic)<br>3. Hispanic Race | Race/Ethnicity<br>3. Mexican American<br>3. Other Hispanic<br>1. Non-Hispanic White<br>2. Non-Hispanic Black<br>2. Other Race / Multi-Racial | 1. White (non-Hispanic)<br>2. Non-White (non-Hispanic)/ Other<br>3. Hispanic Race |
| Birth Place of Mother | Birth Place of Mother<br>1. USA<br>2. Mexico<br>3. Outside USA or Mexico | Female household ref's country of birth<br>1. USA<br>2. Mexico<br>3. Other Spanish speaking country<br>3. Other non-Spanish speaking country | 1. USA<br>2. Mexico<br>3. Outside USA or Mexico |
| Homeowner status | Homeowner<br>1. Yes<br>2. No | Homeowner<br>1. Owned or being bought<br>2. Rented<br>2. Other arrangement | 1. Homeowner<br>2. Not a homeowner |
| Sex of child | 1. Male<br>2. Female | 1. Male<br>2. Female | |
| Maternal age | Maternal age in years | Age of Female household – participant's age (in years) | |
| Child's age | Child's age in months | Participant's age in months | |
| Year of birth | Year of birth as provided | Estimated year of birth: 2nd year of NHANES cycle – participant's age (in years) | |

Abbreviations: USA= United States of America; GED= General education diploma; AA=Associate of Arts; ref=Reference; CHARGE= Childhood Autism Risks from Genetics and the Environment; NHANES= the National Health and Nutrition Examination Survey

**Figure S1**. Flowchart of participant inclusion and exclusion criteria for CHARGE and NHANES samples.

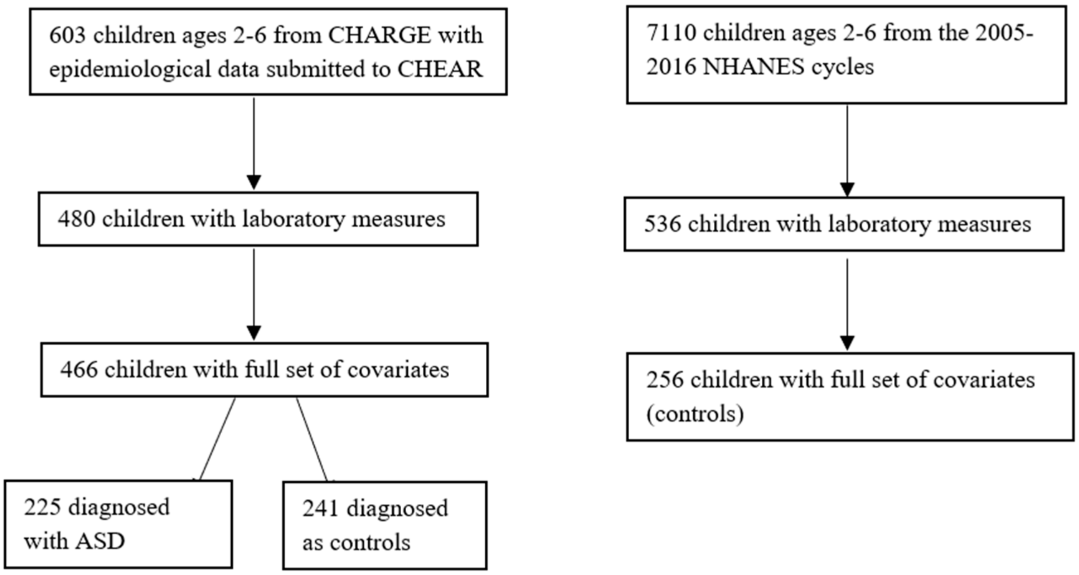

Supplement: Supplementary file 1 [file ijerph-19-02549-s001.zip › ijerph-1567395-supplementary.pdf]
